# Snail promotes metastasis of nasopharyngeal carcinoma partly by down-regulating TEL2

**DOI:** 10.1186/s40880-018-0328-6

**Published:** 2018-09-25

**Authors:** Yi Sang, Chun Cheng, Yi-Xin Zeng, Tiebang Kang

**Affiliations:** 1 State Key Laboratory of Oncology in South China, Collaborative Innovation Center for Cancer Medicine, Sun Yat-sen University Cancer Center, No. 651 Dongfeng East Road, Guangzhou, 510060 People’s Republic of China; 2 Department of Center Laboratory, The Eighth Affiliated Hospital of Sun Yat-Sen University, No. 3025 Shennan Middle Road, Shenzhen, 518033 People’s Republic of China; 3 Jiangxi Key Laboratory of Cancer Metastasis and Precision Treatment, The Third Affiliated Hospital of Nanchang University, No.128 Xianshan North Road, Nanchang, 330008 People’s Republic of China

**Keywords:** TEL2, Snail, Metastasis, Nasopharyngeal carcinoma

## Abstract

**Background:**

Metastasis is the major cause of treatment failure in patients with nasopharyngeal carcinoma (NPC). We previously reported that TEL2, a negative regulator of SERPINE1, could inhibit NPC metastasis to lymph nodes.

**Method:**

A series of in vivo and in vitro assays were performed to elucidate the regulation between Snail and TEL2. TEL2 expression was analyzed in three representative NPC cell lines expressing low levels of Snail (S26, 6-10B, HK1) and two cell lines expressing high levels of Snail (S18, 5-8F). Luciferase and chromatin immunoprecipitation assays were used to analyze the interaction between Snail and TEL2. The roles of the Snail/TEL2 pathway in cell migration and invasion of NPC cells were examined using transwell assays. Metastasis to the lungs was examined using nude mouse receiving NPC cells injection through the tail vein.

**Results:**

Ectopic Snail expression down-regulated TEL2 at the mRNA and protein levels, whereas knockdown of Snail using short hairpin RNA up-regulated TEL2. Luciferase and chromatin immunoprecipitation assays indicated that Snail binds directly to the TEL2 promoter. Ectopic Snail expression enhanced migration and invasion of NPC cells, and such effects were mitigated by TEL2 overexpression. TEL2 overexpression also attenuated hypoxia-induced cell migration and invasion, and increased the number of metastatic pulmonary nodules. Snail overexpression reduced the number of metastatic pulmonary nodules.

**Conclusions:**

TEL2 is a novel target of Snail and suppresses Snail-induced migration, invasion and metastasis in NPC.

**Electronic supplementary material:**

The online version of this article (10.1186/s40880-018-0328-6) contains supplementary material, which is available to authorized users.

## Background

Nasopharyngeal carcinoma (NPC) is highly prevalent in southern China and Southeast Asia [1–4]. In 2013, 42,100 new cases of NPC were diagnosed in China, with 21,320 NPC-related deaths [5]. Estimated rate of metastasis is 15%–30% despite of the exquisite sensitivity to radiation therapy [6, 7]. At the time of diagnosis, 74.5% of the patients have regional lymph node metastasis; 19.9% of the patients have distant metastasis, most often to the liver and lungs [2]. Distant metastasis is the major cause of treatment failure [8–10].

TEL2 (also referred to as ETV7) is a member of the ETS transcription factor family, and plays a key role in hematopoiesis [11, 12]. In a previous study, we identified an inverse relationship between TEL2 expression and metastasis potential in NPC cell lines [2]. We also showed EL2 inhibits NPC metastasis by binding to the promoter of SERPINE1 and down-regulating its expression [2].

Snail is a master regulator of epithelial-to-mesenchymal transition (EMT). High Snail expression is associated with high metastatic potential in colon cancer [13], liver cancer [14, 15], lung cancer [16], as well as NPC by our previous study [13]. Based on these findings, we speculate that TEL2 is a down-stream effector of Snail.

The results of the current study indicated that Snail could down-regulate TEL2 via direct binding to promoter of the TEL2 gene. Consistent with such an action, Snail enhanced NPC cell migration invasion in cultured NPC cells and promoted metastasis to the lungs in nude mice carrying NPC xenograft.

## Methods

### Cell lines

The in vitro experiments were carried out in three representative NPC cell lines expressing low levels of Snail (S26, 6-10B, HK1) and two representative cell lines expressing high levels of Snail (S18, 5-8F) [2–4]. Cells were cultured in Dulbecco’s modified Eagle’s medium (DMEM; Invitrogen) supplemented with 10% fetal bovine serum (FBS; Gibco). All cell lines were authenticated using short-tandem repeat profiling within 6 months prior to the experiments. All five cell lines were obtained from Sun Yat-sen University Cancer Center (SYSUCC).

### Plasmids

The full-length cDNAs of human TEL2 and Snail were cloned into pBABE-puro vector (Cell Biolabs, INC). An HA tag was inserted to the N-terminus of TEL2. The fusion protein Snail-T2A-TEL2, in which Snail and TEL2 were linked by a T2A linker, was also expressed using the pBABE-puro vector. Mutations were introduced using the Quick-Change Site-Directed Mutagenesis Kit (Stratagene), and verified with DNA sequencing.

### Antibodies

Antibody against human TEL2 (Dilution, 1:1000) was obtained from Sigma (HPA029033). Antibodies against human Snail were obtained from R&D (AF3639) for chromatin immunoprecipitation (ChIP) assays and from Cell Signaling Technology (#3895) for Western blotting. Anti-E-cadherin antibody was obtained from BD Company. Antibodies against HA and Tubulin were obtained from Cell Signaling Technology. Antibody against human SERPINE1 was obtained from Santa Cruz (sc-5297).

### RNA interference

Cell lines stably expressing short hairpin RNA (shRNA) targeting Snail transcripts or negative control scrambled shRNA were established using kits from Sigma. The sequences of the 2 human Snail shRNAs are: 5′-ATGCTCATCTGGGACTCTGTC-3′ and 5′-TGCTCCACAAGCACCAAGAGT-3′. Sequence of the short interfering RNA (siRNA) targeting human TEL2 is: 5′-GCCAGATGTGAAGCTCAAATTA-3′.

### RNA extraction and qRT-PCR

These procedures were performed as described previously [3, 17, 18]. Briefly, total RNA was isolated using Trizol reagent (Invitrogen). First-strand cDNA was synthesized using the Revert Aid™ First Strand cDNA Synthesis Kit (MBI Fermentas). The primers were used to amplify target sequences (Additional file 1: Table S1).

### Migration and invasion assays

For transwell migration assays, 1.5 × 10^4^ cells (S18, 5-8F) or 3.5 × 10^4^ cells (S26, 6-10B) in 200 μl of serum-free DMEM were added to cell culture inserts with an 8-μm microporous filter without extracellular matrix coating (Becton–Dickinson Labware). DMEM medium containing 10% FBS was added to the bottom chamber. After 24-h incubation, the cells in the lower surface of the filter were fixed, stained, and examined using a microscope. The numbers of migrated cells from triplicate filters were counted in three random optical fields (×100 magnification) and averaged.

Transwell invasion assays were carried out in the same way, except that the chamber inserts were precoated with Matrigel (Becton–Dickinson Labware).

### Chromatin immunoprecipitation (ChIP) assay

The assay was conducted using a commercial ChIP kit from Active &Motif (cat#: 53040). Briefly, cells were seeded onto 15-cm plates and allowed to grow to 70%–80% confluence. Cells were fixed and collected, and the nuclear pellet was resuspended in ChIP Buffer, subjected to sonication and incubated overnight with 5-μg antibody, followed by incubation with protein G agarose beads for 3 h at 4 °C. DNA–protein complexes were eluted and de-cross-linked through a series of washes. Purified DNA was resuspended in TE buffer and analyzed with PCR using the following primers: E-cadherin-ChIP-F, 5′-ACTCCAGGCTAGAGGGTCACC-3′; E-cadherin-ChIP-R, 5′-CCGCAAGCTCACAGGTGCTTTGCAGTTCC-3′; TEL2-ChIP-E1-F, 5′-TGAATGTGCATTAGTTTATCAAGCC-3′; TEL2-ChIP-E1-R, 5′-CAATCTGCCTACCAGAAATTTATTC-3′; TEL2-ChIP-E2-F, 5′-CACAGTCACGGCTCACTGCAG-3′; TEL2-ChIP-E2-R, 5′-GAGTTGGACACCAGTCTGAACAAC-3′; TEL2-ChIP-E3-F, 5′-GGAGCGCTCAAGACAGAAAGC-3′; TEL2-ChIP-E3-R, 5′-AAAATAGGTTTGGAAATCTAGGTGG-3′; TEL2-ChIP-E4-F, 5′-AGGCAGTAGAGTGGTTAACACAAAC-3′; TEL2-ChIP-E4-R, 5′-TTTATGGAGTTCTCTGTGGATCATG-3′; GAPDH-ChIP-F, 5′-TTCTTGCCTTGCTCTTGCTACTC-3′; and GAPDH-ChIP-R, 5′-AGCCTGCCTGGTGATAATCTTTG-3′.

### Luciferase assay

The assay was carried out as described previously [2, 4]. Briefly, cells were plated in 12-well plates at a density of 2 × 10^5^ per well, and transfected with 0.8-μg promoter-luciferase plasmid. To normalize transfection efficiency, cells were co-transfected with 8-ng pRL-CMV encoding *Renilla* luciferase. After transfection for 48 h, luciferase activity was measured using the Dual-Luciferase Assay kit (Promega). Three independent experiments were performed, and means and standard deviations are presented.

### Animal experiments

Experiments involving animal subjects and all protocols for animal studies were approved by the Research Animal Resource Center of Sun Yat-sen University, in full compliance with the guidelines of the Institutional Animal Care and Use Committee at Sun Yat-sen University Cancer Center. Male athymic mice aged 5–6 weeks were obtained from Shanghai Institutes for Biological Sciences (Shanghai, China). Human NPC cells were resuspended in 100-μl phosphate-buffered saline (PBS; Biological Industries) and injected into the lateral tail vein of mice (3 × 10^6^ cells/animal). At 6 weeks after injection, mice were euthanized. Metastatic nodules were counted with the naked eyes.

### Clinical samples

Experiments involving human tissue samples were approved by the Institutional Review Board of Sun Yat-sen University Cancer Center (YB2015-010). Written informed consent was obtained from all subjects prior to sample collection. Tissue blocks prepared from NPC tissues (10 cases) and lymph node metastases (4 cases) were stored for qRT-PCR.

### Statistical analysis

Differences between two groups were assessed using Student’s *t*-test. Differences among three or more groups were assessed using parametric ANOVA and the least significant difference (LSD) test. Statistical significance was set at *P *< 0.05 (2-sided).

## Results

### Snail down-regulates TEL2

**Fig. 1:**
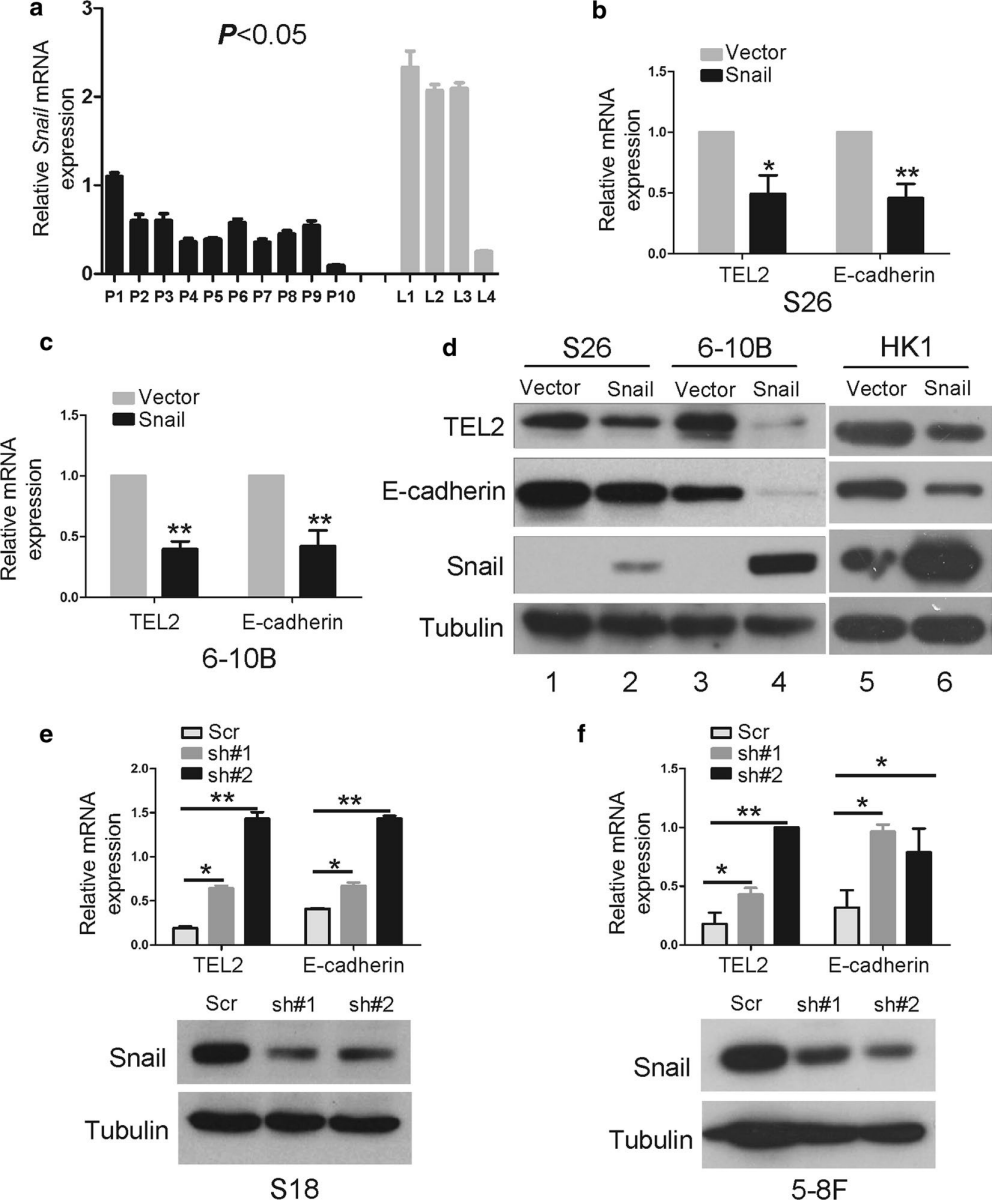
Snail down-regulates TEL2 at the mRNA and protein levels in NPC cells. **a** Levels of Snail mRNA in the indicated tissues. P, NPC primary tissues (*n *= 10); L, metastatic tumor tissues in lymph nodes (*n *= 4). Bars indicate SD. *P < 0.05. **b**, **c** Cells were stably transfected with empty vector or Snail-encoding plasmid, and levels of TEL2 and E-cadherin mRNA were measured using qRT-PCR. Data are mean ± SEM of triplicate samples. *P < 0.05, **P < 0.01. **d** Cells were stably transfected with empty vector or Snail-encoding plasmid, and levels of TEL2 and E-cadherin protein were analyzed by Western blot. **e**, **f** In cells stably transfected with plasmids expressing anti-Snail shRNA or scrambled control shRNA (#1, #2), levels of TEL2 and E-cadherin mRNA were analyzed using qRT-PCR. Data are mean ± SEM of triplicate samples. *P < 0.05, **P < 0.01. Knockdown of Snail at the protein level was confirmed in S18 and 5-8F cells by Western blot

In the present study, we found that levels of Snail mRNA were lower in primary NPC tissues than in metastatic NPC tissues in lymph nodes (Fig. 1a). Stably overexpressing Snail in S26 and 6-10B lines reduced the levels of TEL2 mRNA and protein, with E-cadherin serving as positive control (Fig. 1b–d). In contrast, knockdown of Snail increased TEL2 expression in S18 and 5-8F lines (Fig. 1e, f).

### Snail binds directly to the TEL2 promoter

**Fig. 2:**
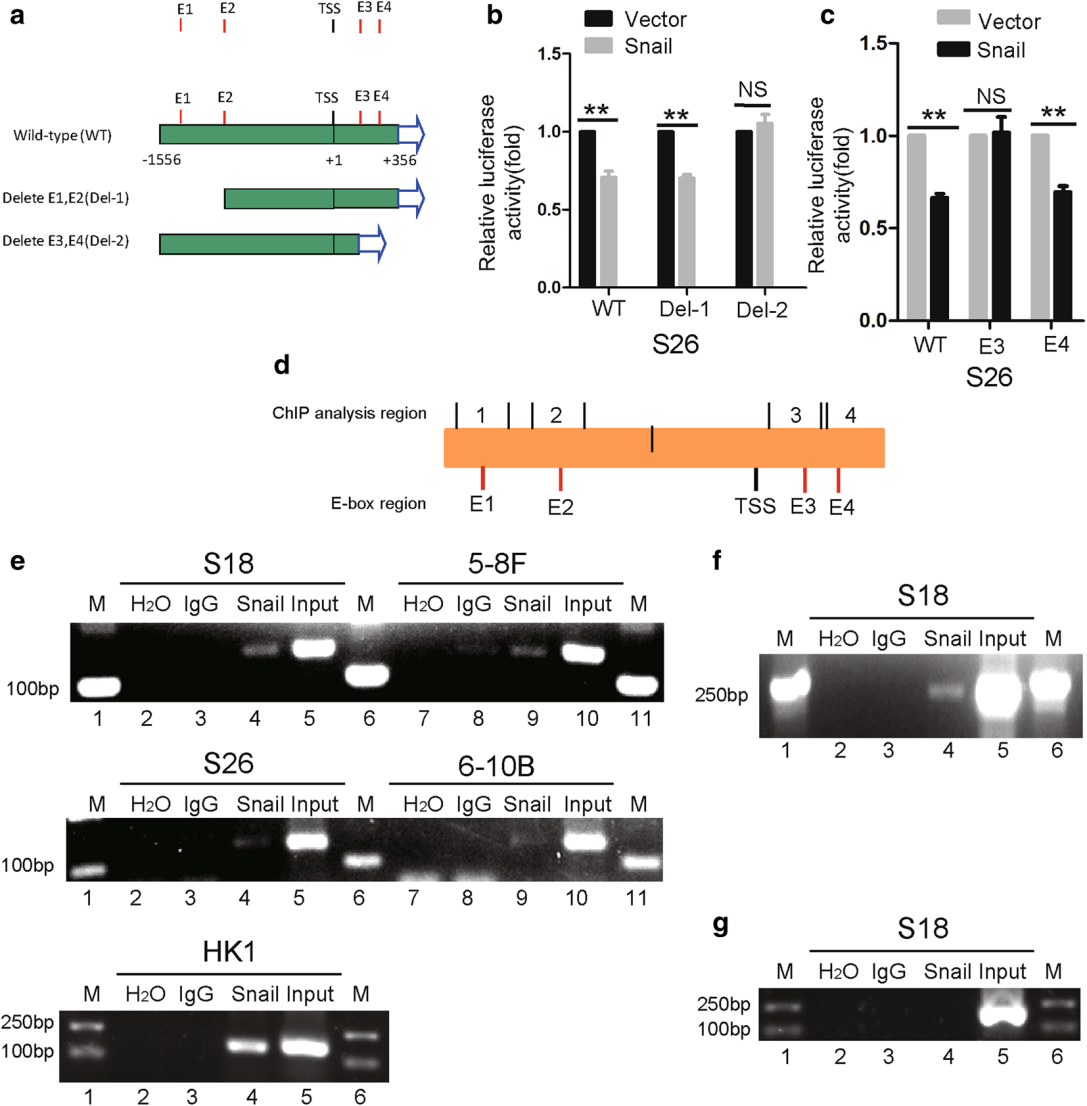
Snail binds directly to the TEL2 promoter in NPC cells. **a** Schematic illustration of the wild-type TEL2 promoter and its mutants in luciferase reporter assays. **b**, **c** S26 cells stably transfected with empty vector or Snail-encoding plasmid were transfected with a luciferase reporter plasmid in which luciferase expression was driven by the wild-type or mutant TEL2 promoter. Luciferase activity was measured as described in Methods. Data are mean ± SEM of triplicate samples. *P < 0.05, **P < 0.01. **d** Schematic illustration of the location of primers for ChIP analysis. **e–g** Cells were analyzed in ChIP assays using anti-Snail antibody as described in “Methods”

In our efforts to investigate how Snail suppresses TEL2 expression in NPC, we analyzed the TEL2 promoter and identified four Snail binding motifs or E-box motifs. We generated several forms of the promoter and tested them in a luciferase reporter assay in order to identify which E-box motifs participate in Snail-mediated suppression of TEL2 (Fig. 2a). Mutation of E3 abolished the suppression of Snail-mediated (Fig. 2b, c). In the ChIP assay, Snail co-precipitated with E3, and this binding was greater in the cell lines S18, 5-8F and HK1 than in cell lines S26 and 6-10B (Fig. 2d, e). Snail did not bind to E1, E2 or E4 in S18 cells (Additional file 1: Figure S1). Throughout these experiments, Snail can bind the promoter of E-cadherin (Fig. 2f). Snail did not bind the promoter of the GAPDH gene (Fig. 2g).

### Snail is involved in hypoxia-induced TEL2 down-regulation

**Fig. 3:**
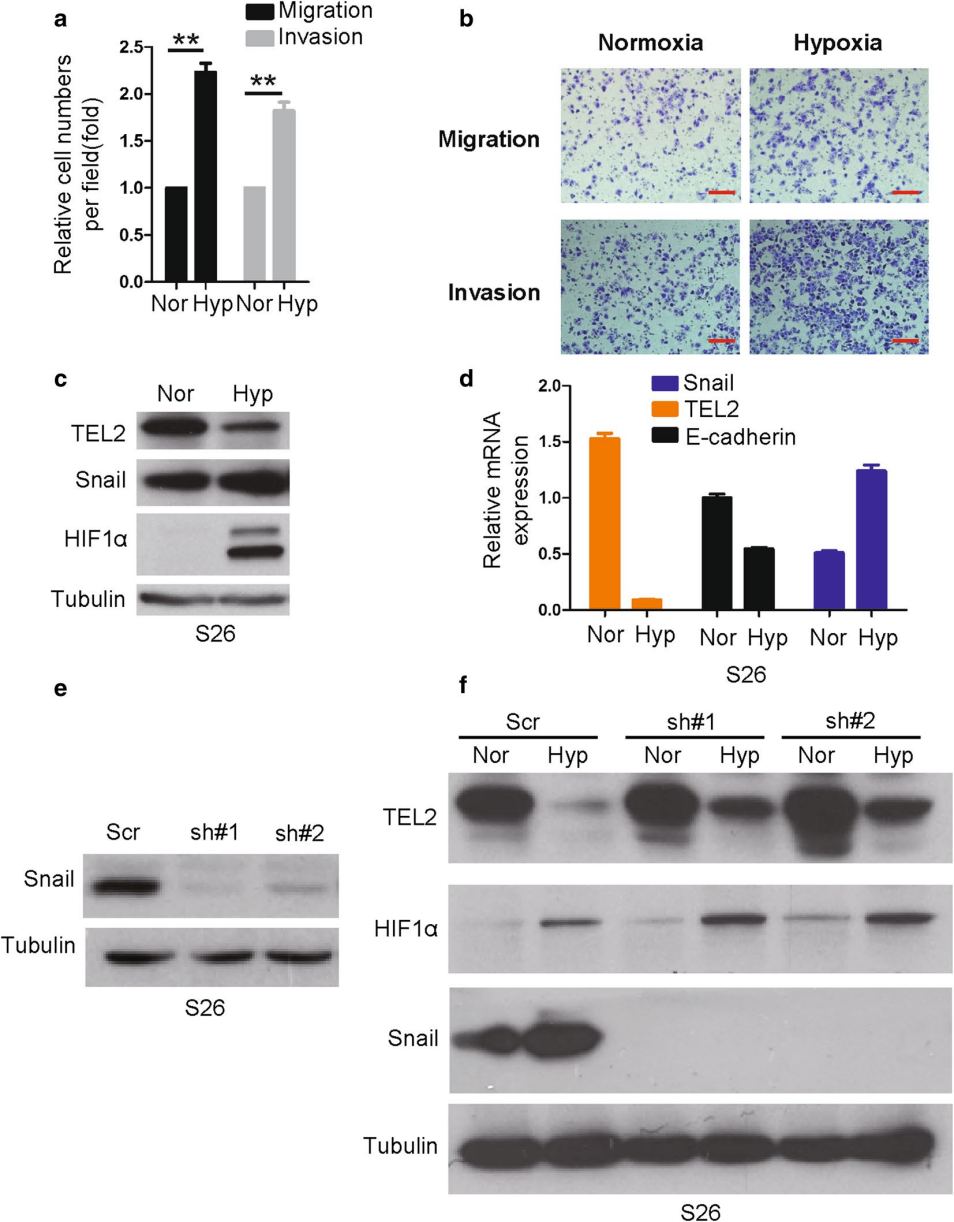
Snail is involved in hypoxia-induced TEL2 down-regulation in NPC. **a** Migration and invasion activity was measured after 24 h in transwell assays under normoxia (Nor, 21% O_2_) or hypoxia (Hyp, 1% O_2_). Data are mean ± SEM. The number of cells passing through the membrane in each transwell was analyzed in triplicate and repeated three times with similar results. **P < 0.01. **b** Representative image of the assay in **a**. Red scale bar, 100 μm. **c** Levels of Snail, HIF1α and TEL2 proteins were analyzed by Western blot in S26 cells exposed for 24 h to normoxia (21% O_2_) or hypoxia (1% O_2_). **d** Levels of Snail, E-cadherin and TEL2 mRNA were measured by qRT-PCR in S26 cells treated as in (**c**). **e** Level of Snail protein in S26 cells was analyzed by Western blot after Snail knockdown. Tubulin was used as a loading control. **f** Levels of TEL2 and HIF1α proteins were measured in the indicated cells after treatment as in **c**. Tubulin was used as a loading control

We found that hypoxia increased migration and invasion of NPC cells in the transwell assay (Fig. 3a, b). As expected, 24-h hypoxia up-regulated Snail and down-regulated TEL2 at the mRNA and protein levels in S26 cells (Fig. 3c, d). This hypoxia-induced TEL2 down-regulation was attenuated by Snail knock-down (Fig. 3e, f).

### A Snail/TEL2/SERPINE1 axis functions

**Fig. 4:**
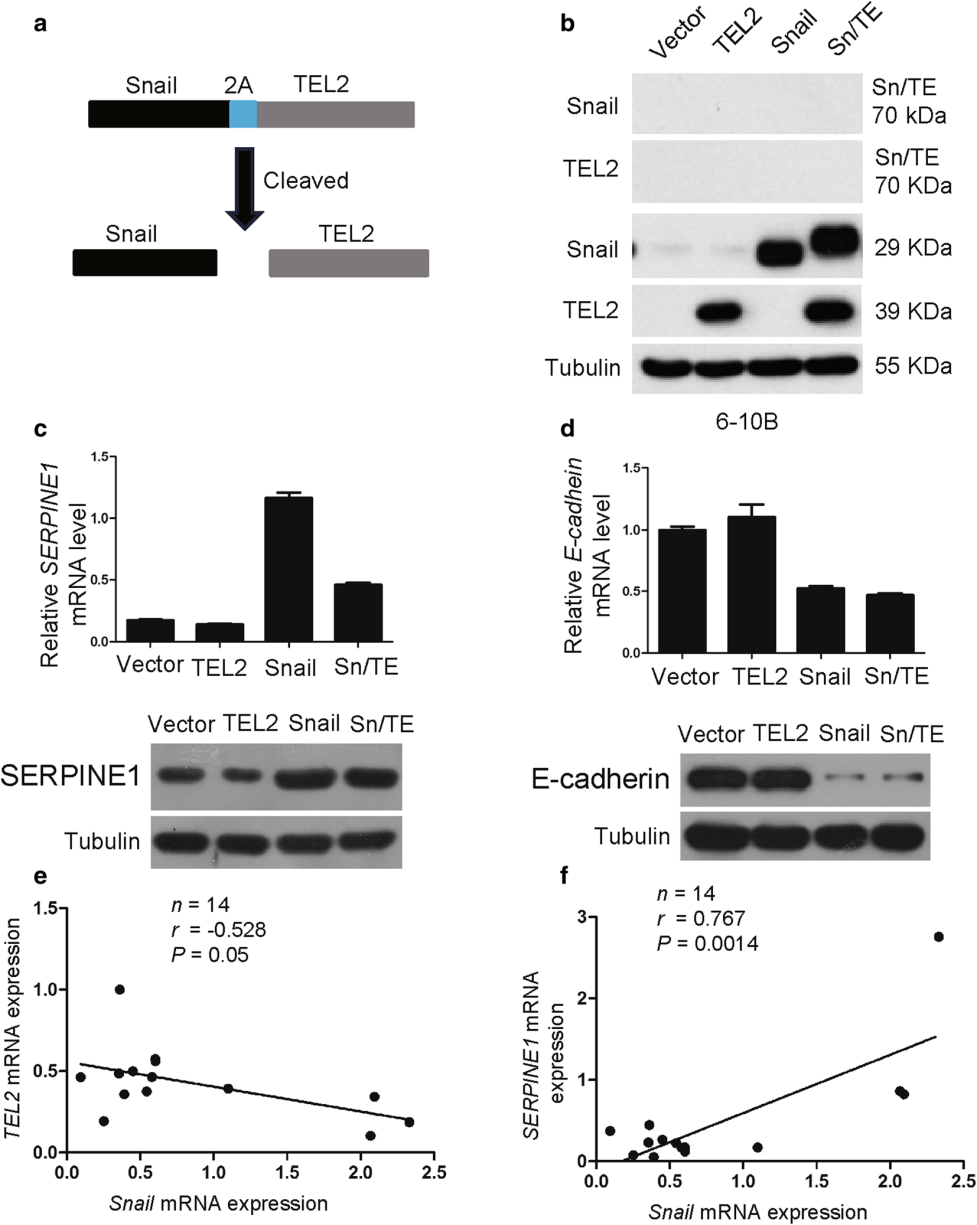
The Snail/TEL2/SERPINE1 axis functions in NPC cells. **a** Schematic illustration for generating a fusion protein of Snail and TEL2 via a T2A linker. **b** Levels of Snail, TEL2 and Snail-TEL2 proteins were determined by Western blot in the indicated cells. Tubulin was used as a loading control. **c** Levels of SERPINE1 mRNA were measured by qRT-PCR and levels of SERPINE1 protein by Western blot. GAPDH served as a control. Data are mean ± SEM of triplicate samples. *P < 0.05, **P < 0.01, ***P < 0.001. **d** Levels of E-cadherin mRNA were measured by qRT-PCR and levels of E-cadherin protein by Western blot. GAPDH served as a control. Data are mean ± SEM of triplicate samples. *P < 0.05, **P < 0.01, ***P < 0.001. **e** In NPC tissues, a significant negative correlation was observed between Snail and TEL2 expression. **f** In NPS tissues, a significant positive correlation was observed between Snail and SERPINE1 expression

To further determine the relationship among Snail, TEL2 and SERPINE1, we fused Snail and TEL2 using a T2A linker, commonly used for fusing multiple coding sequences [19] (Fig. 4a). The resulting fusion protein Snail-T2A-TEL2 (Sn/TE) was cleaved as expected into Snail and TEL2 in stably transfected 6-10B cells (Fig. 4b). SERPINE1 was dramatically overexpressed at the mRNA and protein levels in 6-10B cells stably expressing Snail, but not in cells expressing Sn/TE (Fig. 4c). Co-expression of the two proteins at the Sn/TE rescued the ability of TEL2 to down-regulate SERPINE1. The specificity of these results was confirmed with E-cadherin as negative control: 6-10B cells stably expressing either Snail or Sn/TE showed similar down-regulation of E-cadherin (Fig. 4d). Consistent with these results, we observed a negative correlation between Snail and TEL2 in NPC cells and tissues (Fig. 4e), and a positive correlation between Snail and SERPINE1 in NPC tissues (Fig. 4f).

### Snail promotes NPC cell migration and invasion by down-regulating TEL2

**Fig. 5:**
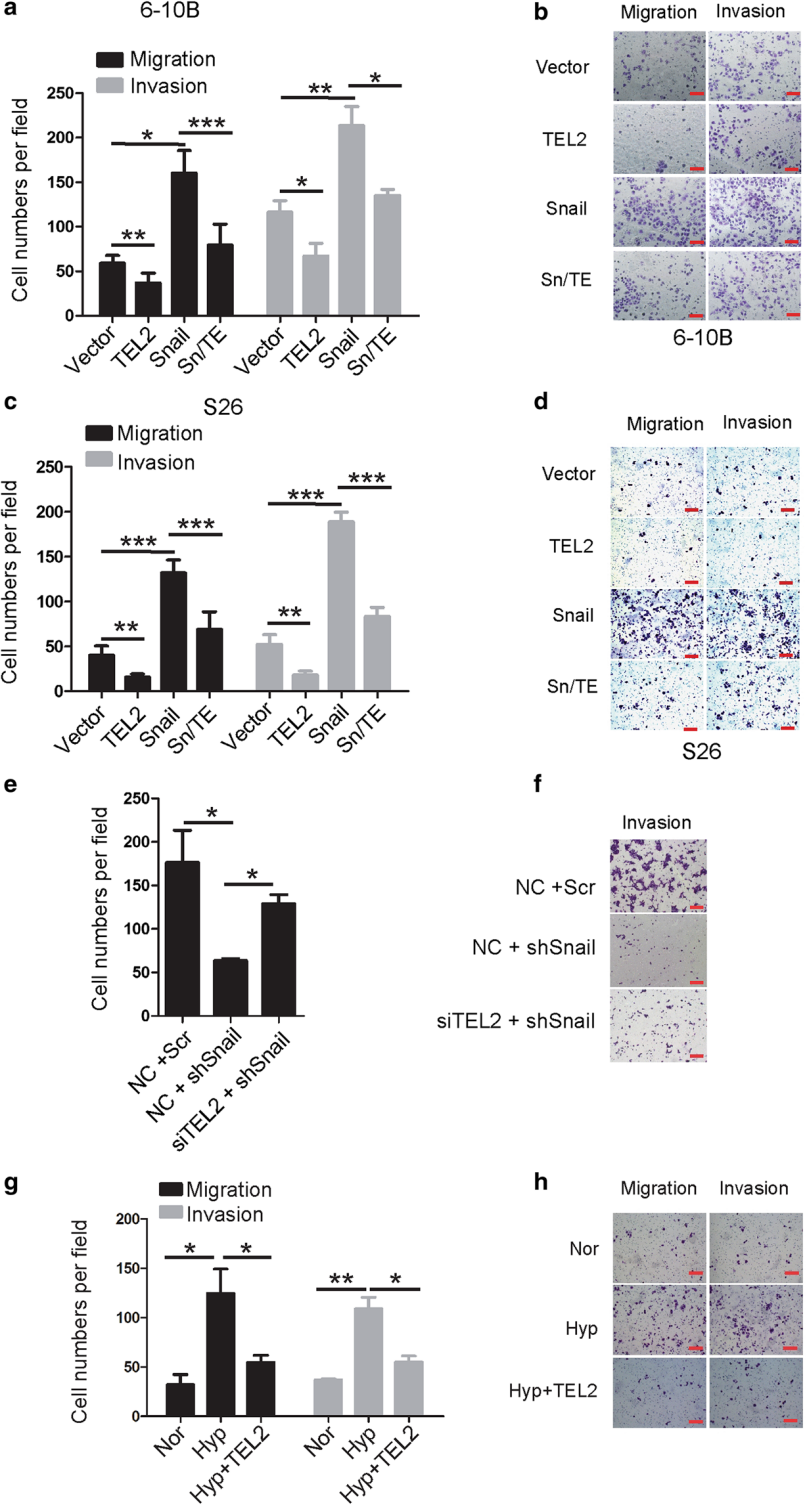
Snail promotes NPC cell migration and invasion mostly by down-regulating TEL2. **a**–**f** Migration and invasion activity of indicated cells were measured at 24 h in transwell assays. The number of cells passing through the membrane in each well was analyzed in triplicate and repeated three times with similar results. Data are mean ± SEM. *P < 0.05, **P < 0.01, ***P < 0.001. **b**, **d**, **f** Representative images of transwell assays. Red scale bar, 100 μm. **e**, **f** The migration and invasion abilities of S26 cells with NC+scr, NC+shSnail or siTEL2+shSnail. The number of cells passing through the membrane in each well was analyzed in triplicate and repeated three times with similar results. Data are mean ± SEM. *P < 0.05, ** *P* < 0.01. **f** Representative image of the transwell assays described in **e**. Red scale bar, 100 μm. **g**, **h** TEL2 overexpression attenuated hypoxia-induced migration and invasion in S26 cells. **h** Representative image of the transwell assays described in **g**. Red scale bar, 100 μm

Ectopic expression of Snail increased the migratory and invasive abilities of NPC cells (Additional file 1: Figure S2). These effects were significantly reduced when TEL2 was overexpressed (Fig. 5a–d). Co-transfecting Snail-deficient cells with siRNA targeting TEL2 and shRNA targeting Snail partly rescued Snail-mediated cell invasiveness (Fig. 5e, f). TEL2 overexpression attenuated hypoxia-induced migration and invasion (Fig. 5g, h).

### Snail promotes NPC metastasis by down-regulating TEL2

**Fig. 6:**
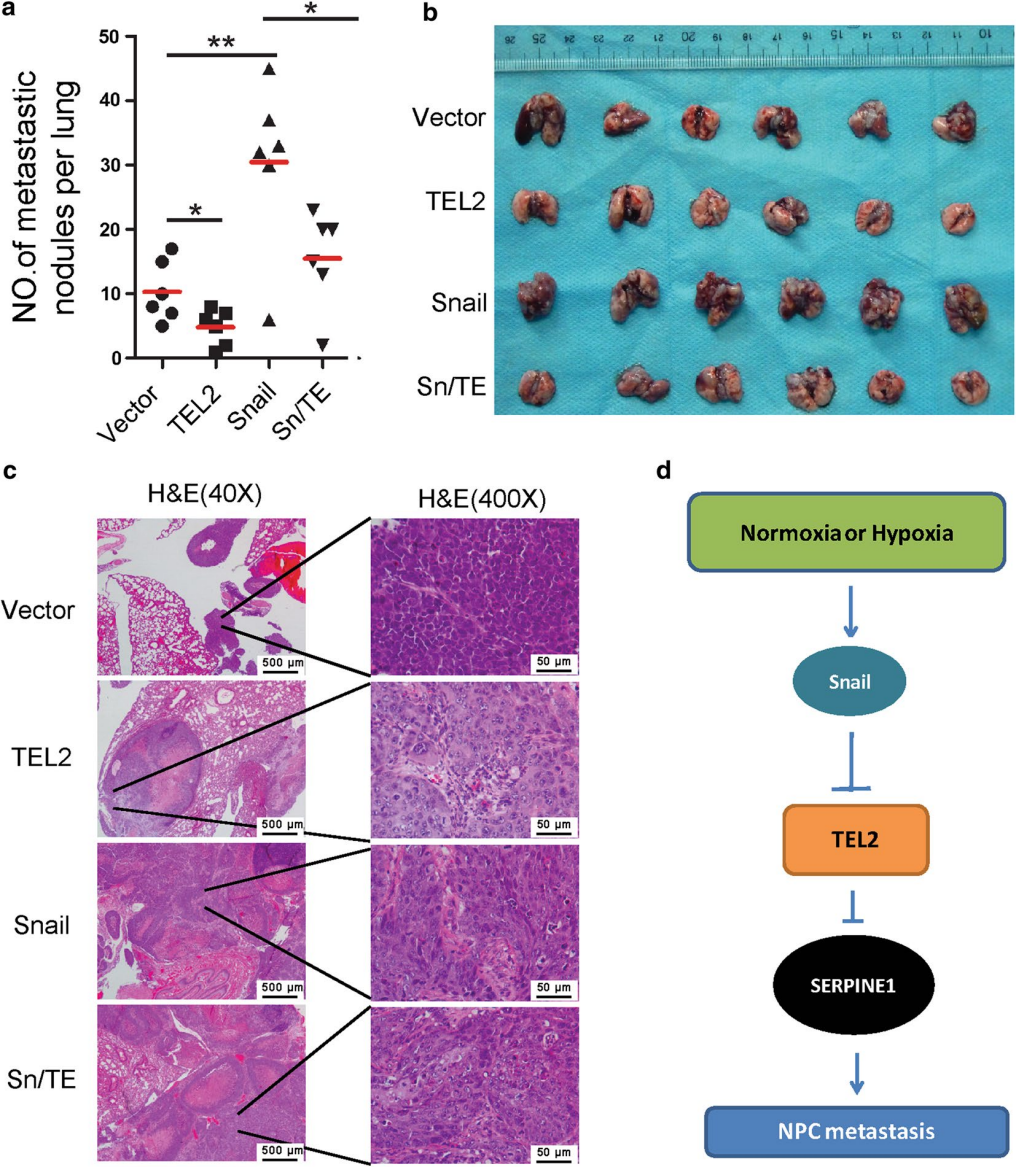
Snail promotes NPC metastasis to lung mostly by down-regulating TEL2 in a nude mouse model. **a**–**c** Stably transfected cells were injected into the lateral tail vein of nude mice. **a** Quantitation of the number of metastases. Data are mean ± SEM (*n *= 6 per group). **b** Macroscopic appearance of metastatic lung tumors. **c** Tumor cross sections with hemotoxylin-eosin staining. Scale bars in (**c**): 500 μm (left), 50 μm **(**right). **d** Proposed model for the regulation and function of TEL2 in NPC metastasis. Snail down-regulates TEL2 during exposure to normoxia or hypoxia, up-regulating SERPINE1, which promotes NPC metastasis

TEL2 overexpression was associated with significantly more metastatic pulmonary nodules, while Snail overexpression was associated with significantly fewer nodules (Fig. 6a–c). The mean number of metastatic nodules was higher in 6-10B cells stably expressing Snail than in cells stably expressing Sn/TE (Fig. 6a–c).

## Discussion

The current study showed, for the first time, that TEL2 is a target of Snail in NPC. Snail is a key transcription factor that regulates cancer metastasis mainly by down-regulating E-cadherin, a key player during EMT and the reverse transition of mesenchymal-to-epithelial transition (MET). Our finding that TEL2 is a downstream target of Snail may explain why Snail overexpression up-regulates SERPINE1, as previously reported [20, 21]. Snail down-regulates TEL2, which in turn down-regulates SERPINE1. Results from the current study suggest that the Snail/TEL2/SERPINE1 axis plays a key role in NPC metastasis.

Hypoxia is present in most NPC tumors, and could promote cancer metastasis [22, 23]. Consistent with a previous study reporting Snail induction by hypoxia [23], we showed increased Snail expression and decreased expression of TEL2 and E-cadherin upon hypoxia in cultured NPC cells. We speculate that the same processes occur in NPC tumors and mediate the ability of hypoxia to promote NPC metastasis.

## Conclusions

Snail down-regulates TEL2 in NPC cells and tissues under both normoxic and hypoxic conditions, ultimately leading to up-regulation of SERPINE1, which promotes metastasis (Fig. 6d). This novel pathway may be valuable for designing new treatments for patients with NPC metastasis.

## Additional file

**Additional file 1.** Additional figures and table.
