# Supplementary material for: Snail promotes metastasis of nasopharyngeal carcinoma partly by down-regulating TEL2
Source: Cancer Commun (Lond). 2018 Sep 25;38:58. doi: 10.1186/s40880-018-0328-6 (PMC6156863; doi:10.1186/s40880-018-0328-6)
Supplement: Supplementary file 1 — Additional file 1. Additional figures and table. [file 40880_2018_328_MOESM1_ESM.docx]

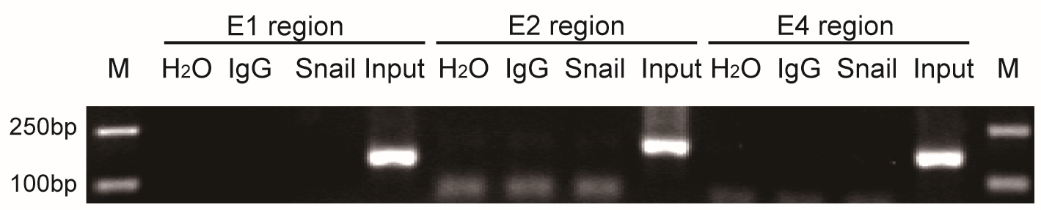


**Additional file 1: Figure S1.** Cells were analyzed in ChIP assays using anti-Snail antibody as described in Methods.


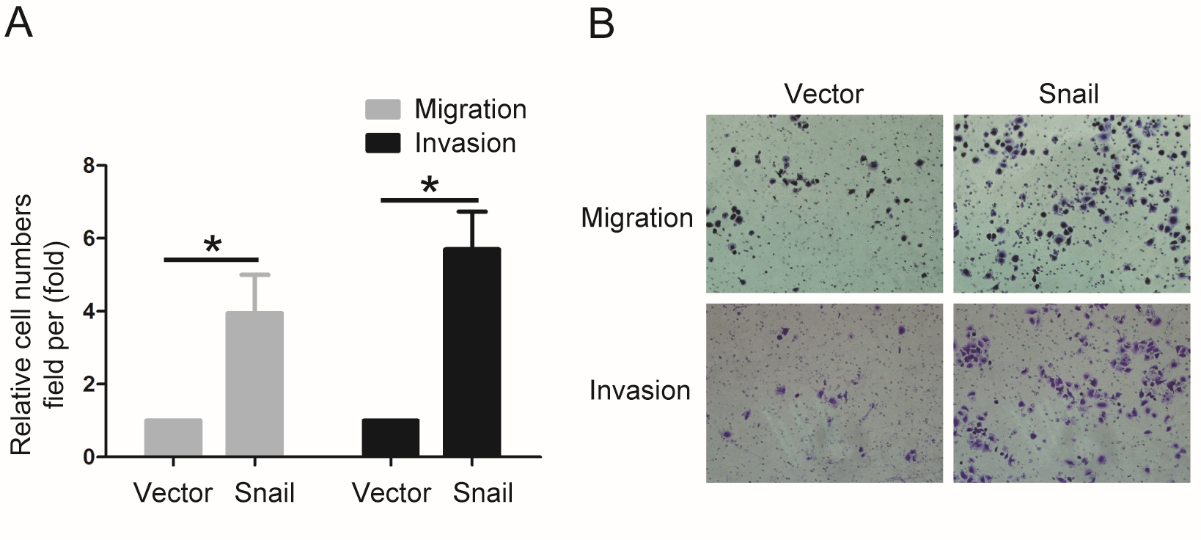


**Additional file 1: Figure S2. Snail promotes migration and invasion by S26 cells.** (**A**)Migration and invasion were measured intranswell assays.The number of cells passing through the membrane in each well was analyzed in triplicate and repeated three times with similar results. Data are mean±SEM values. *P <0.05. (**B)**Representative images of the assays in panel (A).

**Additional file 1: Table S1.**

| Name | Sequence |
| --- | --- |
| TEL2-F | 5’-GGGCTTACCAGCAACTTCG-3’ |
| TEL2-R | 5’-TCTTGGCGTCCTTGTCTTCC-3’ |
| Snail-F | 5’-GTGGGGTTATCTCTGTGTTAGGG-3’ |
| Snail-R | 5’-CCCTGTCCATAG CCTCTACTGC-3’ |
| SERPINE1-F | 5’-AGTGGACTTTTCAGAGGTGGA-3’ |
| SERPINE1-R | 5’-GCCGTTGAAGTAGAGGGCATT-3’ |
| E-cadherin-F | 5’-AATAGTGCCTAAAGTGCTGC-3’ |
| E-cadherin-R | 5’-AGACCCACCTCAATCATCCT-3’ |
| GAPDH-F | 5’-ACAGTCAGCCGCATCTTCTT-3’ |
| GAPDH-R | 5’-GACAAGCTTCCCGTTCTCAG-3’ |
